# Conditional Diffusion Models for CT Image Synthesis from CBCT: A Systematic Review

**DOI:** 10.3390/tomography12050064

**Published:** 2026-05-06

**Authors:** Alzahra Altalib, Chunhui Li, Alessandro Perelli

**Affiliations:** 1 School of Science and Engineering, University of Dundee, Dundee DD1 4HN, UK; c.li@dundee.ac.uk; 2 Department of Applied Medical Sciences, Jordan University of Science and Technology, Irbid 21410, Jordan; 3 School of Cardiovascular and Metabolic Health, University of Glasgow, Glasgow G12 8TA, UK; alessandro.perelli@glasgow.ac.uk

**Keywords:** Cone-beam computed tomography, synthetic CT, tomography, X-ray computed, conditional diffusion models, denoising diffusion probabilistic models, radiotherapy planning, computer assisted

## Abstract

Background: Cone Beam Computed Tomography (CBCT) is widely used in image-guided radiotherapy because it provides on-board volumetric imaging at relatively low doses, but its clinical utility for synthetic CT (sCT) generation remains limited by noise, scatter, artifacts, and reduced Hounsfield Unit (HU) fidelity. Conditional diffusion models (CDMs) have recently emerged as a promising alternative to earlier deep learning approaches because their iterative denoising process may better preserve anatomical structure and model uncertainty. Objective: This systematic review evaluates the use of conditional diffusion models for CBCT-to-CT synthesis, with particular attention to architectural strategies, reported quantitative outcomes, and potential clinical relevance. A systematic search was conducted in PubMed, Web of Science, Scopus, IEEE Xplore, and Google Scholar for studies published between 2013 and 2024. Eleven studies met the eligibility criteria and were analyzed to address three questions: (1) Which conditional diffusion strategies have been used? (2) What outcomes have been reported? and (3) What clinical implications have been discussed? Results: Across the included studies, CDMs frequently showed promising image quality performance, especially when incorporating anatomical priors, spatial-frequency guidance, hierarchical refinement, or latent representations. However, the evidence base remains small and highly heterogeneous with respect to anatomy, dimensionality, supervision strategy, and evaluation metrics, limiting the strength of direct comparative claims. The reviewed literature suggests that conditional diffusion models are a promising direction for CBCT-to-CT synthesis, but stronger dose-aware validation, standardized reporting, and broader multicenter evaluation are still needed before routine clinical deployment. This review has been registered with the International Prospective Register of Systematic Reviews (PROSPERO), under registration number CRD42024619240.

## Simple Summary

Cone Beam Computed Tomography (CBCT) is widely used in radiotherapy because it is fast and relatively low dose, but its image quality is lower than that of conventional CT. Conditional diffusion models have recently been proposed to convert CBCT into synthetic CT with improved anatomical detail and more reliable Hounsfield Units. This systematic review summarizes current CBCT-to-CT synthesis conditional diffusion approaches, their reported quantitative performance, and their possible clinical relevance. The evidence is promising but still limited and heterogeneous, highlighting the need for standardized evaluation, stronger dose-related validation, and larger multi-institutional studies before broad clinical adoption.

## 1. Introduction

Cone Beam Computed Tomography (CBCT) and computed tomography (CT) are two of the widely used methods in clinical settings [1]. The CBCT offers a low-radiation method to provide real-time imaging and is a frequently used technique in image-guided radiotherapy. Despite having numerous applications, it suffers from increased noise, artifacts, and lower contrast for the soft tissues [2]. This may reduce its accuracy for the dose calculations and organ delineation. CT, on the other side, offers higher resolution. This results in a reliable Hounsfield Unit (HU) accuracy and the achievement of better soft tissue contrast. Thus, CT is treated as a gold standard for treatment planning. Nevertheless, CT scans subject patients to higher radiation doses. Thus, patients cannot be subjected to the frequency of exposure to such scans. To cater to these limitations encountered by CT and CBCT, synthetic CT (sCT) generation methods are being used [3]. These methods take advantage of CBCT and CT and transform CBCT to high-quality sCT images, which offer details on par with CT images.

The sCT generation is traditionally carried out using deep learning methods. Two of the most widely used models in this context include generative adversarial networks (GANs) and variational autoencoders (VAEs) [4,5,6]. These methods have shown promising outcomes in the reduction of CBCT artifacts and improving the HU accuracy levels; however, they face some challenges. Some of these challenges include mode collapse, limited structural fidelity, and higher dependency on the paired datasets. Additionally, the complexity inherently associated with medical images, especially that associated with capturing the fine anatomical details and noise, remains a gap to be addressed to attain high structural fidelity. Additionally, these challenges are required to be handled to provide improved generalizability across a diverse range of datasets.

To overcome these limitations, diffusion models have emerged as alternative solutions for the sCT generation [7]. These methods offer several advantages, such as handling the noise, preserving the structural details, and offering improved quantitative accuracy. The diffusion models are based on an iterative refinement strategy for the image denoising. This is followed by a reverse recovery process where images sCT images are generated to be closer to CT images.

This review focuses on the exploration of conditional diffusion models and their applications in the sCT generation. The model evaluation of these methods in light of performance and clinical applications has been carried out. This review focuses on the exploration of conditional diffusion models and their applications in sCT generation. Rather than seeking to establish definitive superiority over earlier approaches, the review aims to synthesize the currently available evidence, describe the main methodological strategies used, and examine the extent to which the reported findings support potential clinical relevance. Given the novelty of the field, particular attention is paid to the limitations of the current evidence base and to priorities for future validation.

From a medical physics perspective, diffusion models offer a probabilistic generative framework that is naturally aligned with uncertainty-aware image reconstruction and dose computation. Unlike deterministic deep learning models that produce a single point estimate, conditional diffusion models learn a conditional distribution over synthetic CT images given CBCT input, enabling voxel-wise variability and uncertainty estimation. This probabilistic formulation is conceptually consistent with established uncertainty propagation principles in radiotherapy, where imaging uncertainties translate into dose calculation uncertainties. As such, diffusion models should not be viewed merely as higher-capacity deep networks but rather as stochastic physical inference models capable of representing ambiguity arising from noise, scatter, and incomplete CBCT information.

## 2. Overview of Diffusion Model Families for CT Image Synthesis

Diffusion-based synthetic CT generation begins with learning a mapping from noisy or artifact-prone CBCT images to clean, diagnostic-quality sCT representations. The target is to recover a high-resolution, anatomically aligned CT image that retains structural fidelity to the CBCT input. Although earlier methods explored GAN-based mappings, their limitations in uncertainty modeling and structural stability have made diffusion-based techniques more attractive.

Diffusion models for CBCT-to-sCT are typically formulated as conditional generative models, where the CBCT serves as a conditioning input. This enables patient-specific image generation with consistent anatomical correspondence. High spatial and contrast fidelity is required to ensure accurate downstream use in the calculation of radiotherapy doses or treatment planning. Thus, generative models must respect both global and local anatomical contexts present in CBCT, although learning to correct for modality-specific limitations such as noise and scatter.

### CT Output Specificity in CBCT-Conditioned Models

The output specificity of sCT generation models is of paramount clinical importance. For sCT to be usable in dose computation or image-guided radiotherapy, the generated CT must not only appear realistic but also be anatomically accurate with respect to CBCT. Diffusion models provide several advantages here:

- Structural Preservation: The denoising process is inherently robust to noise perturbations, and when strongly conditioned on CBCT, the model tends to preserve macro and micro anatomical features, including subtle tissue boundaries.
- Uncertainty Modeling: Unlike deterministic models, diffusion models model a posterior distribution over CT outputs conditioned on CBCT. This allows clinicians to assess confidence levels and potentially detect ambiguous or low-quality regions.
- Multi-modal Consistency: When paired with frequency domain or multi-scale conditioning, such as FGDA, the model can enforce consistency between CT texture and CBCT geometry, thus increasing diagnostic confidence.

Output specificity can be quantitatively assessed using metrics such as structural similarity (SSIM), peak signal-to-noise ratio (PSNR), and clinical dose deviation. However, visual and expert-based evaluations remain crucial due to the clinical significance of subtle anatomical discrepancies.

In summary, strong CBCT conditioning combined with diffusion-based generative modelling yields high-specificity synthetic CT images suitable for integration into clinical workflows.

## 3. Analysis of Diffusion Model Variants

In the following sections, we analyze four prominent diffusion model variants—denoising diffusion probabilistic models (DDPMs), denoising diffusion implicit models (DDIMs), latent diffusion models (LDMs), and frequency-guided diffusion models (FGDMs)—within the context of CBCT-conditioned synthetic CT generation. Our exposition emphasizes both the theoretical and clinical implications.

### 3.1. Denoising Diffusion Probabilistic Models (DDPMs)

DDPMs constitute the foundational class of diffusion models. They simulate a forward process in which Gaussian noise is gradually added to an image over multiple time steps and a reverse process that learns to denoise this corrupted data iteratively, ultimately recovering the clean image distribution from noise [8]. The DDPM approach is formally grounded in variational inference and models the data likelihood through a series of conditional Gaussians.

Let $x_{0}\in R^{H\times W}$ denote the ground truth CT image and let y be the conditioning CBCT input image. The goal is to learn a conditional distribution $p_{\theta }\left(x_{0}|y\right)$ that generates synthetic CT images $x_{0}$ conditioned on y.

A DDPM defines a denoising process *q* that gradually adds Gaussian noise to the data in *T* steps. The process is defined as

(1) $$\begin{array}{ccc} q(x_{1:T}|x_{0}) & = & \prod_{t=1}^{T}q\left(x_{t}|x_{t-1}\right),\\ q(x_{t}|x_{t-1}) & := & N(x_{t};\sqrt{1-\beta_{t}}x_{t-1},\beta_{t}I) \end{array}$$

where ${\left\{\beta_{t}\right\}}_{t=1}^{T}$ is a variance schedule, typically linear or cosine. The marginal distribution of $x_{t}$ given $x_{0}$ can be derived analytically

(2) $$q\left(x_{t}|x_{0}\right)=N\left(x_{t};\sqrt{{\bar{\alpha }}_{t}}x_{0},\left(1-{\bar{\alpha }}_{t}\right)I\right),$$

where $\alpha_{t}:=1-\beta_{t}$ and ${\bar{\alpha }}_{t}:=\prod_{s=1}^{t}\alpha_{s}$. This allows one to sample $x_{t}∼q\left(x_{t}|x_{0}\right)$ directly.

The reverse (generative) process is another Markov chain parameterized by a neural network

(3) $$\begin{array}{ccc} p_{\theta }\left({\hat{x}}_{0:T}|y\right) & = & p\left(x_{T}\right)\prod_{t=1}^{T}p_{\theta }\left({\hat{x}}_{t-1}|{\hat{x}}_{t},y\right),\\ p_{\theta }\left({\hat{x}}_{t-1}|{\hat{x}}_{t},y\right) & := & N({\hat{x}}_{t-1};\mu_{\theta }\left({\hat{x}}_{t},t,y\right),\Sigma_{\theta }\left({\hat{x}}_{t},t,y\right)) \end{array}$$

In most implementations, the variance $\sum_{\theta }$ is either fixed or learned separately. A common simplification uses

(4) $$\mu_{\theta }\left({\hat{x}}_{t},t,y\right)=\frac{1}{\sqrt{\alpha_{t}}}\left({\hat{x}}_{t}-\frac{\beta_{t}}{\sqrt{1-{\bar{\alpha }}_{t}}}\epsilon_{\theta }\left({\hat{x}}_{t},t,y\right)\right),$$

where $\epsilon_{\theta }$ predicts the noise added at step *t*.

The training loss is based on the variational lower bound (VLB) on the conditional negative log-likelihood which is

(5) $$\log p_{\theta }\left(x_{0}|y\right)\geq E_{q}\left[\log\frac{p_{\theta }\left(x_{0:T}|y\right)}{q(x_{1:T}|x_{0})}\right]=:-L_{\mathrm{VLB}}$$

This decomposes into per timestep KL divergences

(6) $$L_{\mathrm{VLB}}=E_{q}\left[\sum_{t=1}^{T}D_{\mathrm{KL}}\left(q(x_{t-1}|x_{t},x_{0})\;\|\;p_{\theta }\left({\hat{x}}_{t-1}|{\hat{x}}_{t},y\right)\right)-\log p_{\theta }\left({\hat{x}}_{0}|{\hat{x}}_{1},y\right)\right]$$

A practical surrogate loss simplifies the training objective to

(7) $$L_{\mathrm{simple}}=E_{x_{0},\epsilon ,t}\left[{∥\epsilon -\epsilon_{\theta }\left(\sqrt{{\bar{\alpha }}_{t}}x_{0}+\sqrt{1-{\bar{\alpha }}_{t}}\epsilon ,t,y\right)∥}^{2}\right]$$

where ϵ∼N(0,I). This is essentially denoising score matching.

**Figure 1:**
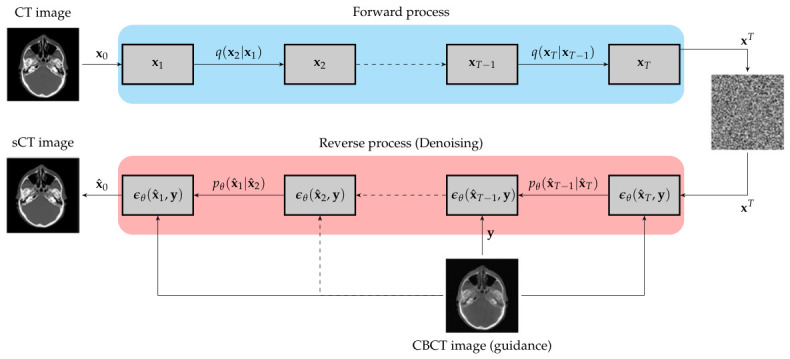
Workflow of Conditional DDPM for CBCT to CT image synthesis.

This approach has been depicted in Figure 1 and allows them to reconstruct high-quality sCT images with improved artefact reduction and HU accuracy.

At inference, to generate an sCT image it involves the reverse generative process as in Algorithm 1.
**Algorithm 1** DDPM algorithm**Require:** Sample $x_{T}∼N\left(0,I\right).$   1:**for** t=T,…,1 **do**   2:    ${\hat{x}}_{t-1}∼N\left(\mu_{\theta }\left({\hat{x}}_{t},t,y\right),\Sigma_{t}\right)$**Output:** $x_{0}$   3:**end for**

This process is stochastic and allows for sampling multiple plausible sCTs per input y.

### 3.2. Conditioning Mechanisms in CBCT-to-sCT Diffusion Models

As mentioned above, conditioning the diffusion process on CBCT input is central to the success of sCT generation. The goal is to guide the generative process so that the output is not only plausible as a CT image but also accurately reflects the anatomical structure present in the CBCT. Several strategies have been explored in the literature:

- Input Concatenation: The CBCT volume is concatenated with the noisy latent or image sample at each denoising step. Often it is implemented using a conditional UNet where $\epsilon_{\theta }\left({\hat{x}}_{t},t,y\right)$ takes y as input, typically via concatenation. This direct approach has the benefit of simplicity and early integration of structural information.
- Feature Modulation (FiLM): The CBCT input y is encoded via a convolutional network, and its features are used to modulate the internal layers of the denoising network. FiLM layers apply affine transformations conditioned on CBCT features, allowing spatial and channel-specific influence. This effectively models $p_{\theta }\left(x_{0}|y\right)$ without needing explicit paired supervision.
- Cross-Attention: Particularly powerful in vision transformers and UNet-based diffusion models, cross-attention enables the model to selectively integrate information from CBCT across spatial scales. The attention maps provide interpretability and allow flexible registration-free alignment.
- Classifier-Free Guidance (CFG): This strategy trains the diffusion model with and without conditioning. During inference, guidance strength is controlled by interpolating between the two outputs. This enhances fidelity by adjusting conditional vs. unconditional noise estimates
  (8) $$\epsilon_{\mathrm{guided}}=\left(1+w\right)\epsilon_{\theta }\left({\hat{x}}_{t},t,y\right)-w\epsilon_{\theta }\left({\hat{x}}_{t},t\right),$$
  where w>0 is a guidance scale.
  This allows tuning the influence of CBCT during generation, which is especially useful when dealing with varying levels of CBCT quality.

Each of these mechanisms seeks to achieve anatomical fidelity while allowing flexibility in how much and where CBCT information is used. For sCT applications, high-resolution alignment, especially in bone and soft tissue boundaries, is critical. Thus, architectural choices that facilitate multiscale fusion of CBCT features are preferred.

The advantages of the DDPM are that it captures uncertainty since output diversity reflects aleatoric uncertainty in sCT generation, and it is a non-adversarial training approach, which is more stable compared to GANs. However, the sampling might be slow since it requires T∼1000 and large compute resources for training and inference. Furthermore, it is of utmost importance to align CT and CBCT conditioning features overall. Although DDPMs yield high-fidelity outputs, they are computationally intensive due to the large number of sequential denoising steps required.

### 3.3. Denoising Diffusion Implicit Models (DDIMs)

DDIMs redefine the generative process as a non-Markovian, deterministic mapping that preserves the same training objective as DDPM but modifies the sampling process, allowing fewer sampling steps without significant loss in quality [9]. By leveraging a reparameterized trajectory through the diffusion space, DDIMs enable faster inference.

Given a noisy sample $x_{t}$, we deterministically obtain $x_{t-1}$ via:

(9) $${\hat{x}}_{t-1}=\sqrt{{\bar{\alpha }}_{t-1}}x_{0}+\sqrt{1-{\bar{\alpha }}_{t-1}-\eta^{2}\frac{1-{\bar{\alpha }}_{t}}{1-{\bar{\alpha }}_{t-1}}}\epsilon_{\theta }\left({\hat{x}}_{t},t,y\right)+\eta \epsilon ,$$

where η∈[0,1] controls the stochasticity (η=0 yields a fully deterministic path). In the most common setting:

(10) $${\hat{x}}_{t-1}=\sqrt{{\bar{\alpha }}_{t-1}}\left(\frac{{\hat{x}}_{t}-\sqrt{1-{\bar{\alpha }}_{t}}\epsilon_{\theta }\left({\hat{x}}_{t},t,y\right)}{\sqrt{{\bar{\alpha }}_{t}}}\right)+\sqrt{1-{\bar{\alpha }}_{t-1}}\epsilon_{\theta }\left({\hat{x}}_{t},t,y\right).$$

This eliminates the need to sample Gaussian noise ϵ during generation, drastically accelerating inference; for example, from 1000 to 50 steps, without retraining.

Although not always emphasized in medical imaging tasks, DDIMs provide a practical trade-off between speed and image quality, making them suitable for real-time or interactive applications.

### 3.4. Latent Diffusion Models (LDMs)

Latent diffusion models address the computational burden of pixel space generation by operating in a compressed latent space learned via autoencoders or variational encoders (VAE) [10]. By learning the diffusion process in this low-dimensional domain, LDMs dramatically lower the memory and runtime cost, enabling the use of higher-resolution medical data. Once denoising is completed in the latent domain, a decoder reconstructs the final image. These models are the class of generative models that can work and operate on the low dimensional latent space instead of the direct image space. LDMs make use of VAE-based encoder-decoder settings for learning the compressed latent representation of the CT images. A CT image $x_{0}$ is first encoded into a latent vector $z_{0}$ using a VAE encoder E(·):

(11) $$z_{0}=E\left(x_{0}\right)$$

and the forward diffusion process operates in latent space, where the noise is progressively added to the latent vector $z_{0}$ to obtain a noisy latent vector $z_{t}$ at time step *t*:

(12) $$z_{t}=\sqrt{{\bar{\alpha }}_{t}}z_{0}+\sqrt{1-{\bar{\alpha }}_{t}}\epsilon ,\;\epsilon ∼N\left(0,I\right)$$

In the reverse process, the CT image generation is conditioned on the CBCT image y, which is passed through a condition encoder to generate a context vector used to guide the reverse diffusion

(13) $$z_{y}=E_{y}\left(y\right)$$

This conditional embedding captures anatomical priors for guiding the reverse denoising process.

In the reverse process the noisy latent vector $z_{t}$ at timestep *t*, is used predicts the noise component $\epsilon_{t}$ using the and the condition embedding $z_{y}$:

(14) $$\epsilon_{t}=\epsilon_{\theta }\left({\hat{z}}_{t},t,z_{y}\right)$$

The DDIM style update is then applied

(15) $${\hat{z}}_{t-1}=\sqrt{{\bar{\alpha }}_{t-1}}\left(\frac{{\hat{z}}_{t}-\sqrt{1-{\bar{\alpha }}_{t}}\epsilon_{t}}{{\bar{\alpha }}_{t}}\right)+\sqrt{1-{\bar{\alpha }}_{t-1}}\epsilon_{t}$$

After denoising the latent back to $z_{0}$, the final sCT image is reconstructed using the VAE decoder D(·):

(16) $${\hat{x}}_{0}=D\left({\hat{z}}_{0}\right)$$

**Figure 2:**
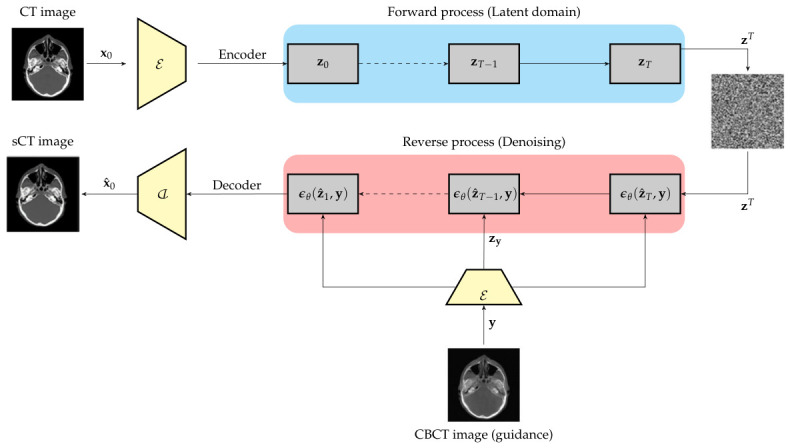
Workflow of LDM for CT image synthesis using CBCT guidance.

The diagram in Figure 2 shows the training workflow of the forward and reverse diffusion processes in the latent domain with conditional CBCT guidance.

### 3.5. Frequency-Guided Diffusion Models (FGDMs)

FGDMs introduce frequency domain priors to diffusion-based generation, with the aim of improving the recovery of high-frequency details often lost in noisy imaging modalities [11]. These models typically embed frequency-aware losses or frequency-decomposed guidance into the diffusion pipeline, enabling sharper reconstruction of anatomical boundaries and fine textures. For CBCT applications, FGDMs are particularly effective in restoring bone edges and soft tissue transitions, which are otherwise blurred due to scatter and noise.

FGDA aids in improving the diffusion process through an incorporation of spatial and frequency domain information. The idea is to denoise the network and frequency domain features that are extracted from the CBCT images. This process leads to improved anatomical details and fidelity in the sCT reconstruction. As in standard diffusion models, the forward process perturbs a clean CT image $x_{0}$ into a noisy version $x_{T}$ by sequentially adding Gaussian noise:

(17) $$x_{t}=\sqrt{{\bar{\alpha }}_{t}}x_{0}+\sqrt{1-{\bar{\alpha }}_{t}}\epsilon ,\;\epsilon ∼N\left(0,I\right),\;t=1,\ldots ,T$$

To guide the reverse process, a frequency domain representation *f* of the image is extracted by applying a frequency transform, DCT or FFT F, to the CBCT image Y to obtain frequency coefficients. A frequency encoder $E_{f}\left(\cdot \right)$ and condition encoder $E_{c}\left(\cdot \right)$ are used to derive rich feature representations. To guide the reverse process, a combined conditioning vector is obtained by concatenating spatial and frequency-based features:

(18) $$z_{f}=E_{f}\left(F\left(y\right)\right)⊕E_{c}\left(y\right)$$

where F(·) denotes the frequency transform and ⊕ indicated the vector concatenation.

The reverse process is carried out using a noise predictor $\epsilon_{\theta }$ guided by both spatial and frequency information:

(19) $$\epsilon_{t}=\epsilon_{\theta }\left({\hat{x}}_{t},t,z_{f}\right)$$

This is used in the deterministic update rule (as in DDIM):

(20) $${\hat{x}}_{t-1}=\sqrt{{\bar{\alpha }}_{t-1}}\left(\frac{{\hat{x}}_{t}-\sqrt{1-{\bar{\alpha }}_{t}}\epsilon_{t}}{{\bar{\alpha }}_{t}}\right)+\sqrt{1-{\bar{\alpha }}_{t-1}}\epsilon_{t}$$

**Figure 3:**
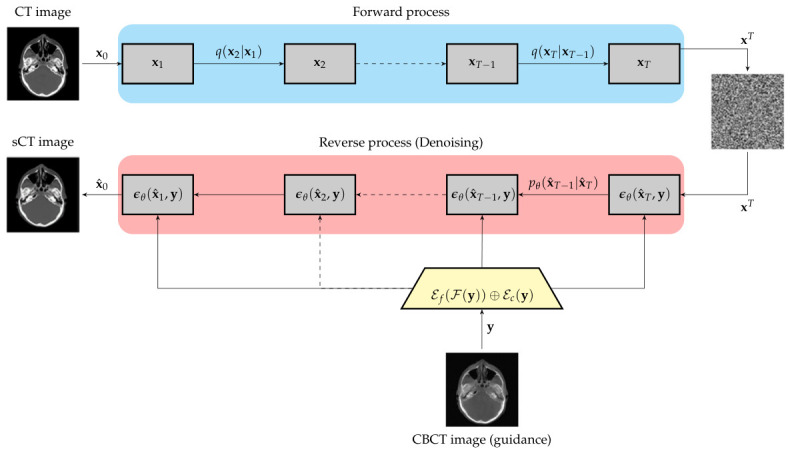
Workflow of the Frequency Guided Diffusion model for CT image Synthesis.

The diagram in Figure 3 shows the training workflow of the diffusion process with conditional CBCT frequency guidance.

### 3.6. Comparison with Prior Reviews

Several reviews and surveys have previously examined sCT generation for radiotherapy; however, their scopes and emphases differ considerably from the present work. Ref. [12] provided a broad overview of deep-learning-based sCT generation across multiple modalities (MRI, PET, and CBCT), focusing on convolutional and adversarial networks, but without dedicated attention to diffusion-based methods. Ref. [13] presented a systematic review that included MRI → CT, CBCT → CT, and PET attenuation correction tasks; while CBCT-based synthesis was included, it formed only one component of a wider multi-modal evaluation, and diffusion approaches were not yet a main focus. Our earlier article [14] conducted a PRISMA-guided review dedicated to synthetic CT generation from CBCT, summarizing CNN-based, GAN-based, and early transformer approaches. At that time, conditional diffusion models were either absent from the literature or mentioned only briefly as an emerging direction. In contrast, the present manuscript focuses exclusively on conditional diffusion models for CBCT-to-CT synthesis. This narrower scope enables a more detailed examination of conditioning strategies, architectural design choices, training paradigms, and specific radiotherapy applications that cannot be addressed in a comprehensive review. As diffusion models have become the most rapidly advancing class of generative techniques in medical imaging, this targeted systematic review addresses a distinct and timely research question that was not covered in our earlier publication. Additionally, the *SynthRAD2023 Challenge Report* [15] benchmarked MRI → CT and CBCT → CT models across institutions, highlighting the weak correlation between image-based similarity metrics and dosimetric accuracy, an observation that further motivates the inclusion of dose-relevant outcomes in the present review. The summary of the works mentioned is listed in Table 1.

Compared with these works, the novelty of the current review lies in its exclusive focus on conditional diffusion models for CBCT → CT synthesis, offering: (i) a dedicated taxonomy of conditional diffusion architectures (noise conditioning, guidance strategies, latent vs pixel space design); (ii) a structured synthesis of quantitative performance relative to CNN/GAN baselines; (iii) a cross-study analysis that integrates image similarity and dose-based accuracy, bridging algorithmic and clinical relevance. This work therefore complements broader sCT reviews by narrowing the lens to diffusion-based CBCT → CT translation and providing both methodological clarity and clinical context to this rapidly evolving domain.

## 4. Aim of the Study

The primary aim of this systematic review is to critically evaluate the application of conditional diffusion models for generating sCT images from CBCT data. Specifically, this study seeks to address the limitations of CBCT, such as image noise, scatter, and artifacts, by exploring how diffusion models can improve the quality and clinical utility of sCT. The review aims to systematically identify the methodologies employed in conditional diffusion approaches, compare their performance with traditional deep learning techniques in terms of accuracy and robustness, and examine their clinical relevance. Because this is a rapidly developing field with emerging literature, the systematic review contains a limited number of published studies. Ultimately, the study aims to provide insights into the potential of conditional diffusion models to enhance dosimetric precision and anatomical fidelity in radiotherapy, although highlighting gaps and directions for future research.

### Research Questions

1. Which conditional diffusion strategies have been used for sCT generation from CBCT?
2. What quantitative and clinically relevant outcomes have been reported for these models, and how do they compare descriptively with comparator approaches across studies?
3. What methodological and translational limitations currently constrain the clinical applicability of conditional diffusion models for sCT generation?

## 5. Methodology

This section begins by outlining the search strategy employed to identify relevant studies for the review. Following this, a comprehensive description of the systematic and methodical steps undertaken to conduct the review is provided, ensuring transparency and replicability of the research process.

### 5.1. Search Strategy

This systematic review followed the PRISMA 2020 statement [16] and used the PICO framework in Table 2 to guide search development. The literature search was conducted across PubMed, Web of Science, Scopus, IEEE Xplore, and Google Scholar for studies published between 2013 and 2024. Search terms combined controlled vocabulary, where supported, with free text keywords related to diffusion-based generative modeling and CBCT-to-CT synthesis. Core concepts included “diffusion model”, “conditional diffusion”, “denoising diffusion”, “score-based generative model”, “cone-beam computed tomography”, “CBCT”, “synthetic CT”, and radiotherapy-related terms. Database-specific Boolean search strings and search limits are provided in Supplementary Table S1 to ensure reproducibility. Searches were limited to English-language publications. Because this is a rapidly developing field, screening also considered emerging literature indexed in the searched databases. The handling of conference proceedings and preprints is stated explicitly in the eligibility criteria below.

### 5.2. Inclusion Criteria

1. Articles explicitly addressing diffusion models for synthetic CT generation.
2. Research employing conditional approaches in diffusion models, such as guidance by specific features or anatomical priors.
3. Peer-reviewed journal articles, conference proceedings, and directly relevant preprints were eligible if they reported sufficient methodological detail and unique information relevant to the review question; non-peer-reviewed records were explicitly flagged in the synthesis and interpreted with additional caution.
4. Publications from the last 11 years (2013–2024).
5. Articles published in English.

### 5.3. Exclusion Criteria

1. Studies do not involve diffusion models as a primary method for synthetic CT generation.
2. Papers on CBCT enhancement, noise reduction, or artifact correction unrelated to synthetic CT generation.
3. Non-peer-reviewed blogs, editorials, or opinion pieces.
4. Duplicates across databases.
5. Articles lacking sufficient methodological details or evaluation metrics.
6. Articles published in non-English languages or without reliable translations.
7. Publications before 2013 unless they are foundational studies in diffusion models.

### 5.4. Study Selection

Following the systematic search, articles are screened based on their titles and abstracts to identify potentially relevant studies. After undergoing an initial screening process, articles proceed to a full-text review, where their suitability for inclusion in the systematic review is thoroughly assessed. The inclusion/exclusion criteria were applied strictly during the screening process, with reasons for exclusion documented for transparency and reproducibility.

### 5.5. Data Extraction

Following the retrieval of results from the database search, the identified records were imported into a reference management tool, EndNote, to organize and consolidate the search outcomes. During this process, duplicate entries were systematically identified and removed. The subsequent screening of studies was conducted independently by two reviewers, Alzahra Altalib (AA) and Alessandro Perelli (AP), who applied the predefined eligibility criteria to determine the suitability of studies for inclusion in the systematic review. Any disagreements between the reviewers were resolved through discussion to achieve consensus. Data extraction was performed from the final set of selected articles using a standardized approach. Key details were collected, including publication information (e.g., year, authors, and country of origin) and specific parameters related to diffusion models, such as noise injection methods, dataset types, and model architectures, etc. The extracted study-level dataset used for this review is provided in Supplementary Table S2 to improve transparency and reproducibility.

### 5.6. Quality Assessment

To assess the methodological quality of the included studies, two researchers, AA and AP, employed the Quality Assessment of Diagnostic Accuracy Studies-2 (QUADAS-2) tool, as outlined by [17]. This tool was specifically used to evaluate the risk of bias and ensure methodological rigor. By providing a structured framework, QUADAS-2 facilitated a systematic assessment of potential biases, the overall quality, and the robustness of each study included in the systematic review.

### 5.7. Data Synthesis

The syntheses were examined and are presented to identify general patterns, advantages, limitations, and deficiencies in the research pertaining to the use of diffusion models for sCT generation. The information extraction was based on pre-defined criteria that have been presented later in Table 3. Because the included studies differed substantially in anatomical region, dataset composition, paired versus unpaired supervision, dimensionality, comparator models, and reported outcome measures, formal quantitative cross-study comparison was not considered methodologically appropriate. We therefore performed a structured descriptive synthesis. Studies were compared at the study level according to conditioning strategy, anatomy, dimensionality, supervision paradigm, and reported quantitative or clinically proximal outcomes. Heterogeneity was treated as a central consideration in interpretation rather than being reduced to a pooled comparative estimate.

## 6. Results

**Figure 4:**
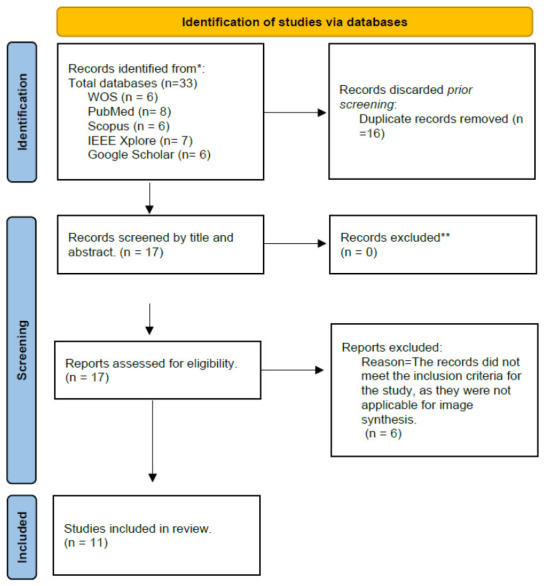
PRISMA flow diagram of conditional diffusion models for CBCT to CT image synthesis, summarizing identification, screening, exclusion, and inclusion of studies. * Records identified from each database before duplicate removal. ** Title and abstract screening was performed manually without automation tools; no records were excluded at this stage.

The database search yielded a total of 33 records, distributed as follows: 6 from Web of Science (WoS), 8 from PubMed, 6 from Scopus, 7 from IEEE Xplore, and 6 from Google Scholar. After the removal of duplicate entries, 17 unique records remained. Then these records were assessed according to predetermined inclusion and exclusion criteria. As a result, 6 records were excluded because they did not meet the study’s inclusion criteria, specifically because they were not applicable to image synthesis. Consequently, 11 records were included in this systematic review. The screening process is fully described in PRISMA Figure 4.

The articles included and reviewed as part of this study collectively explored advanced methods for sCT generation using CBCT and CT images. In all of these reviewed studies, emphasis has been kept on the use of the diffusion model to achieve images with improved quality, and artifact reduction and to be suitable for clinical settings. Numerous approaches have been presented, including frequency-guided diffusion models (FGDM) [18], stacked coarse-to-fine architectures [23], and patient-specific fine-tuning [7,19]. These works propose to address the inherent limitations associated with CBCT images, such as noise, artifacts, and inaccuracies associated with Hounsfield Unit (HU) values. The studies incorporated a diverse range of datasets that included paired and unpaired CBCT-CT image slices, dual-energy CT (DECT) scans, etc. These scans have been developed for the context of radiation therapy and proton therapy [24]. The study further incorporated numerous loss functions, including frequency domain regularization [18], edge-aware constraints [26], and hybrid condition losses [7,23]. These loss functions help preserve anatomical details while achieving structural and dosimetric accuracy.

The proposed neural network architectures, including Swin-UNETs [24], dual-branch attention modules [26], and texture-preserving frameworks [26], were reported to achieve promising performance in terms of PSNR, MAE, and SSIM within their respective study settings. However, direct comparison across studies remains limited by substantial heterogeneity in anatomy, supervision strategy, dimensionality, dataset composition, and reported endpoints. The current literature, therefore, supports the view that diffusion-based models are promising but does not yet justify strong cross-study claims of consistent superiority over earlier GAN- or VAE-based approaches. Despite these advancements, challenges such as computational inefficiency, dependency on paired datasets, and limited robustness to frequency variations persist [18,23,26]. Overall, the findings highlight the transformative potential of diffusion-based models for improving CBCT-to-CT synthesis. The studies have found improvements in adaptive radiotherapy, proton therapy planning, and broader clinical applications [18,19,24]. A detailed synthesis of the included articles has been presented in Table 3.

### Study Characteristics and Outcome Reporting

The 11 included studies were heterogeneous across several key dimensions. Anatomical targets included lung, head and neck, brain, pelvis, pancreas, prostate, chest tumor, and multicenter mixed CBCT-CT cohorts. Both paired and unpaired supervision strategies were represented, and models were implemented in 2D, 3D, and latent-space formulations. Outcome reporting also varied considerably: some studies emphasized MAE and PSNR, others reported SSIM, gamma passing rate, or dose-related findings, and some studies presented partial or architecture specific outcomes only.

This heterogeneity has two implications. First, the field cannot currently be summarized reliably using a single pooled quantitative estimate. Second, interpretation should distinguish methodological promise from generalizable evidence. Accordingly, the remainder of the Results section is organized as a descriptive synthesis of reported outcomes and heterogeneity patterns rather than a ranked quantitative comparison across studies.

## 7. Quantitative Analysis

**Figure 5:**
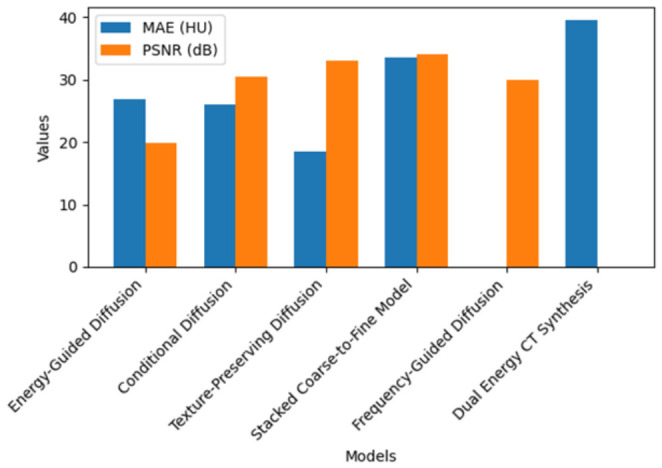
MAE and PSNR achievement for the reviewed models.

The quantitative outcomes reported in the included studies are summarized descriptively in this section. Because the studies differed substantially in anatomy, supervision strategy, dimensionality, dataset composition, and reported endpoints, these results are presented as study-level observations rather than as a formal pooled comparison. The most commonly reported image quality metrics were MAE and PSNR, with a smaller number of studies additionally reporting SSIM, gamma passing rate, or dose-related findings. As shown in Figure 5, several included studies reported favorable MAE and PSNR values within their respective experimental settings. For example, the texture-preserving diffusion model reported an MAE of 18.48 HU and a PSNR of 33.07 dB, while the conditional diffusion model reported an MAE of 25.99 HU and a PSNR of 30.49 dB. These findings suggest promising image quality performance, although direct cross-study comparison remains limited by heterogeneity.

**Figure 6:**
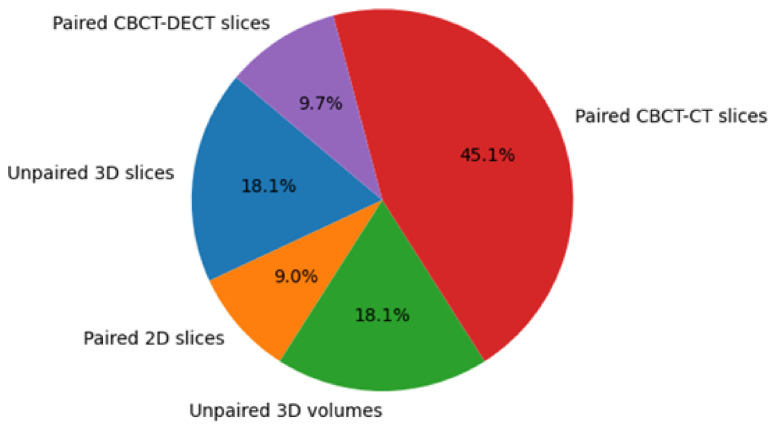
Data sampling and distribution across models.

The included studies also varied substantially in dataset size and structure, as illustrated in Figure 6. This variation reflects the diversity of current experimental settings rather than a directly comparable benchmark. For example, the stacked coarse-to-fine model was evaluated on a paired CBCT-CT pelvic dataset of 250 patients, whereas the conditional diffusion model was evaluated on a smaller 50-patient brain and head-and-neck cohort. Frequency-guided diffusion models were reported in both paired and unpaired settings, illustrating the diversity of current experimental designs. However, the presence of different supervision regimes further limits direct comparison of reported performance across studies.

**Figure 7:**
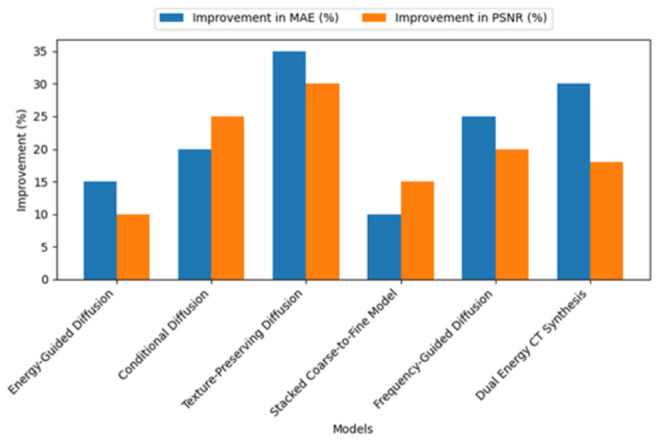
Reported study-specific percentage improvements relative to comparator models, where such comparisons were explicitly provided in the original articles. These comparisons are descriptive due to heterogeneity in anatomy, datasets, supervision strategies, and baseline models.

Where individual studies explicitly reported percentage improvements relative to their own comparator models, these results are summarized in Figure 7 for descriptive context only. However, these comparisons are study-specific and should not be interpreted as evidence of uniform superiority across the field, because the included studies used different anatomies, datasets, supervision paradigms, and baseline methods. However, based on the available studies, we can report that diffusion models have been found to improve the MAE and PSNR performance compared to GANs and VAEs. The texture-preserving diffusion model has been found to achieve an improvement of 35% in MAE and a 30% gain in PSNR. Similarly, Frequency-Guided Diffusion has achieved a 25% improvement in MAE and a 20% gain in PSNR. Only a minority of the included studies reported clinically proximal endpoints beyond image-similarity metrics. Most studies primarily emphasized MAE and PSNR, whereas fewer reported SSIM, gamma passing rate, or dose-related relevance. For example, one latent diffusion study reported a gamma passing rate of 93.8%, while other studies discussed dosimetric accuracy or treatment planning relevance in adaptive radiotherapy settings. However, these clinically meaningful endpoints were reported inconsistently and with different evaluation frameworks, which limits direct synthesis of translational benefit. Overall, the current evidence suggests potential clinical value, but systematic dose-aware validation remains limited.

## 8. Discussion

The review highlights the potential of conditional diffusion models in sCT generation. A central finding of this review is that heterogeneity, rather than simple average performance, currently defines the evidence base. For this reason, the present synthesis should be interpreted as a structured qualitative/descriptive review of an emerging literature rather than as a basis for formal quantitative cross-study ranking. The diffusion models have been found to address the challenges associated with low-quality CBCT images in the form of artifacts, noise, and structural inaccuracies. The use of diffusion models is relatively unexplored; however, the reviewed models include FGDM, Texture-Preserving Diffusion Models, and Stacked Coarse-to-Fine Models as being the most dominant and promising methods. These methods rely on the use of advanced loss functions such as frequency domain regularization and edge-aware constraints. This helps them to retain the fine anatomical details and to improve the dosimetric accuracy. The use of datasets across the studies further highlights the adaptability of the models across several clinical settings. The dataset approach ranges from single-institution paired datasets and multicenter unpaired datasets. The FGDM and Texture-Preserving Diffusion have further illustrated the MAE and PSNR performance and were found to improve sCT image quality by reducing the artifacts. Compared to the traditional deep learning models, including GANs and VAEs, the diffusion models are outperforming. This is valid in terms of structural fidelity and quantitative metrics. For example, the Texture-Preserving Diffusion model has shown an MAE improvement of 35% and a PSNR improvement of 30%. This is potentially due to the iterative refinement approach adopted by the models that render high-quality images by integrating domain-specific priors and noise reduction. However, some of the challenges remain to be addressed, including the computational requirement and high reliance on the paired datasets. This may somehow limit them from becoming ubiquitous in clinical settings. In clinical settings, the use of diffusion models for sCT generation can have significant implications in terms of adaptive radiotherapy and proton therapy planning. The models work by reducing the artifacts and improving HU accuracy, thereby leading to improved dose computations and precise treatment planning. This is especially true for anatomically challenging regions. Models like FGDM can also work with unpaired datasets to facilitate scalability.

### 8.1. Clinical Relevance and Practical Considerations

Recent approaches based on diffusion models have shown improvement in the qualitative accuracy of synthetic CT images. However, their clinical relevance depends on their quantifiable impacts on radiotherapy workflows. The next step in the clinical approach is to make a direct correlation between the enhanced image quality and treatment accuracy in the dose calculation. Thus, integration of conditional diffusion models into clinical pipelines can reduce uncertainty in CBCT-based dose computation. This can enable more precise dose delivery and adaptive re-planning. In addition, future research shall emphasize the scalability and inference efficiency to ensure real-time applicability in online adaptive radiotherapy. Domain adaptation strategies include fine-tuning across different scanners, protocols, and anatomical sites. These will be essential to maintain consistent performance in heterogeneous clinical environments. Such considerations highlight the importance of bridging technical advancement and having measurable radiotherapy outcomes to fully realize the translational potential of diffusion-based CBCT to CT synthesis.

In particular, future studies should directly evaluate whether improvements in sCT image quality translate into more accurate dose calculation, more reliable treatment planning, and measurable clinical benefit in adaptive radiotherapy workflows. At present, this remains a key limitation of the literature, because clinically meaningful endpoints were reported in only a subset of the included studies and were not evaluated using standardized criteria across the field.

In addition to image quality and artifact suppression, studies have reported promising clinical implications of sCT synthesis for radiotherapy applications. For instance, diffusion-based sCT generation has been evaluated for its impact on dose reconstruction accuracy. This shows a mean dose difference below 2% compared to planning sCT in head-and-neck and pelvic cases. Such improvements translate into more reliable adaptive re-planning and target coverage assessment. In addition, the high-fidelity sCT outputs can facilitate accurate auto contouring of organs at risk. These can reduce manual workload and variability. Preliminary investigations into workflow integration have shown the feasibility of incorporating trained diffusion models into online adaptive pipelines. These can have an inference time of less than 5 to 10 s per volume on modern GPUs.

### 8.2. Answer to Research Questions

The three research questions identified as part of this work in the introduction have been answered as follows:

1. The conditional diffusion models for sCT generation are limited yet have adopted several approaches. These include Frequency-Guided Diffusion Models, Texture-Preserving Diffusion Models, and Stacked Coarse-to-Fine Models. The models are based on advanced loss functions, including frequency domain regularization, edge-aware constrained losses and hybrid conditional losses. This helps with ensuring that high anatomical accuracy is achieved and artifacts are reduced. In addition, some adoptive techniques, such as dual-mode feature fusion and hierarchical learning, are also found in the articles reviewed.
2. Diffusion models have typically been found to outperform traditional deep learning models. These specifically include GANs and VAEs in terms of accuracy. Quantitative assessment has shown that models consistently achieve low MAE and high PSNR. The best-performing model, Texture-Preserving Diffusion, has shown a 35% improvement in MAE and a 30% improvement in PSNR performance compared to GANs. Such improvements are due to the adoption of an iterative approach using domain-specific priors. This allows diffusion models to handle the noise and artifacts effectively.
3. The clinical implications of diffusion models for sCT generation are expected to be numerous. By enabling a reduction of artifacts and showing a tendency to increase HU accuracy, diffusion models can allow precise dose calculations. This may lead to improved treatment planning in adaptive radiotherapy and proton therapy. The ability of the models to work with unpaired datasets (FGDM, for instance) helps with improved scalability and thus applicability in many clinical settings. Overall, diffusion models have shown the potential to improve patient outcomes through safe and effective radiation treatments. However, further validations are needed to analyze the computational inefficiencies associated with these models.

### 8.3. Risk of Bias Assessment

**Figure 8:**
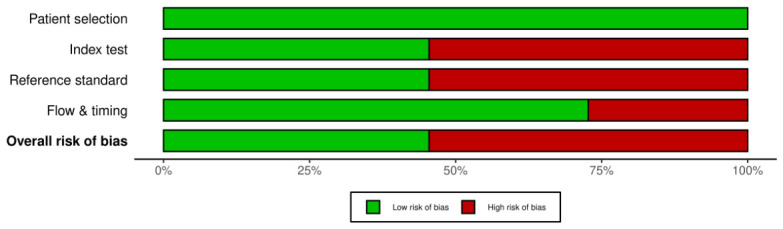
The risk of bias assessment results for all the included studies was conducted using QUADAS 2.

The QUADAS-2 assessment showed some variability in methodological quality across the included studies. All studies were rated as low concern in patient selection, indicating generally acceptable dataset selection procedures. lower overall concern was observed in [11,18,23,25,26], which were rated Low across all four domains and therefore had Low overall concern. In contrast, refs. [7,19,20,21,22,24] showed high overall concern, mainly due to limitations in index tests and reference methods, and in some studies also flow and timing. These findings indicate that, although quantitative results were often favorable, the strength of evidence was not uniform across studies. Greater confidence may therefore be placed in studies with low overall concern, whereas results from studies with high overall concern should be interpreted more cautiously. Because the included evidence showed mixed methodological quality, the findings were synthesized qualitatively rather than through formal meta-analysis. These results are summarized in Figure 8.

### 8.4. Registration and Reporting

The findings of this systematic review were reported in adherence to the Preferred Reporting Items for Systematic Reviews (PRISMA) guidelines. The review was conducted in accordance with a pre-established protocol registered with the International Prospective Register of Systematic Reviews (PROSPERO), bearing the registration number CRD42024619240. To improve transparency and reproducibility, the Supplementary Materials, Table S1, provide the database-specific search strategies; Table S2 provides the extracted study-level dataset used in the descriptive synthesis; and Figure S1 provides the QUADAS-2 risk of bias (traffic lights).

### 8.5. Hounsfield Unit Accuracy and Dose Calculation Sensitivity

In radiotherapy treatment planning, errors in synthetic CT Hounsfield Unit (HU) values propagate nonlinearly into electron density and stopping power estimation, directly affecting dose calculation accuracy. Prior physics studies have demonstrated that systematic HU deviations of approximately 30–50 HU in soft tissue can result in dose calculation errors on the order of 1–3%, depending on beam energy, tissue composition, and anatomical location. In high-gradient regions or heterogeneous anatomies such as the lung or head and neck, spatially structured HU errors extending over several centimeters may lead to even larger localized dose deviations than uncorrelated voxel-wise noise of similar magnitude. Although most diffusion-based sCT studies primarily report image similarity metrics such as MAE or PSNR, the reported MAE values in the range of 15–30 HU fall within a regime generally considered acceptable for photon radiotherapy planning. However, the clinical relevance of these metrics ultimately depends on the spatial distribution of residual HU errors rather than their global average, underscoring the need for dose-aware evaluation strategies.

In the context of this review, this is particularly relevant because most included studies reported global image similarity metrics, whereas only a smaller subset evaluated clinically proximal outcomes that more directly reflect treatment planning impact.

### 8.6. Uncertainty Quantification in Diffusion-Based sCT

A defining advantage of diffusion models over conventional deterministic networks is their ability to generate multiple plausible realizations of synthetic CT for a single CBCT input. From a medical physics standpoint, this enables explicit estimation of aleatoric uncertainty arising from imaging noise and scatter. Voxel-wise uncertainty can be quantified using the variance or credible intervals computed across multiple diffusion samples, providing spatial maps of prediction confidence. Such uncertainty estimates are particularly relevant in radiotherapy, where regions of elevated HU uncertainty may correspond to increased dose calculation sensitivity. For example, uncertainty envelopes on stopping power ratios could be propagated through dose engines to estimate dose volume histogram (DVH) variability. Despite this potential, none of the reviewed studies performed formal uncertainty propagation into dose metrics, highlighting a critical gap between probabilistic image synthesis and physics-based treatment planning.

### 8.7. Scanner and Protocol Variability

CBCT acquisition parameters vary substantially across institutions and vendors, including tube voltage (typically 80–140 kVp), bowtie filtration, detector geometry, and scatter correction algorithms. These factors can introduce systematic HU shifts exceeding 100 HU in CBCT images, particularly in low density or high scatter regions, while diffusion models demonstrate robustness to random noise, their ability to correct protocol dependent bias remains insufficiently characterized. Several reviewed studies employed single institution datasets or fixed acquisition settings, limiting conclusions regarding cross scanner generalizability. Without explicit modeling of scanner variability, diffusion-based sCT models may inadvertently learn site specific correction patterns that fail to generalize, which is of particular concern for multi center adaptive radiotherapy workflows.

## 9. Future Directions: Physics-Informed and Dose Aware Diffusion Modeling

Future diffusion-based sCT frameworks could benefit from tighter integration with physical constraints relevant to radiotherapy. Physics-informed diffusion models may incorporate explicit priors on electron density continuity, mass density conservation, or material segmentation consistency. Additionally, dose-aware training strategies could be explored, where loss functions penalize HU errors weighted by local dose sensitivity rather than uniform voxel-wise differences. For example, HU deviations in regions contributing minimally to dose deposition may be clinically less relevant than small errors near target volumes or organs at risk. Incorporating differentiable dose engines or surrogate dose models into the diffusion training loop could align image synthesis objectives with downstream clinical impact, bridging the gap between image realism and treatment accuracy.

## 10. Conclusions

In conclusion, the advancement in the diffusion models, including conditional and denoising diffusion approaches, has been found to exhibit high performance for sCT generation from CBCT images. The model helps bridge the gaps with the traditional models in terms of noise handling and achieving high structural fidelity. The diffusion models have been found to outperform traditional models, including GANs and VAEs, exhibiting higher MAE, PSNR, and SSIM performance. These models rely on an iterative refinement approach with domain-specific priors. This enables them to extract accurate image synthesis, as well as dose calculations and treatment planning. This is especially true in adaptive radiotherapy and proton therapy. Despite these benefits, some of the validations need to be performed, including the analysis of computational inefficiencies and the experimentation of real-world clinical data (especially unpaired). Future research shall focus on the optimization of these models for their clinical scalability and to ensure their robust performance in the intersubject domain. In summary, diffusion models are found to have promising outcomes in radiotherapy outcomes and have the potential to improve patient care through precise and reliable imaging. Overall, this review provides the first comprehensive synthesis focused solely on conditional diffusion models for CBCT → CT synthesis. Through a structured taxonomy and quantitative comparison with conventional CNN and GAN approaches, it clarifies the present capabilities and limitations of diffusion-based image translation. The findings highlight the potential of these models to enhance anatomical fidelity and dose consistency while underscoring the need for standardized evaluation protocols and larger, multi-institutional validation to enable clinical deployment. Future validation should therefore focus on establishing direct links between image quality improvements and downstream radiotherapy endpoints, including dose calculation accuracy, treatment planning robustness, and broader clinical utility.

## Figures and Tables

**Table 1:** Comparison of existing reviews and the present work.

Reference	Scope Modalities	Architectures Covered	CBCT → CT Focus	Diffusion Models Discussed	Evaluation Emphasis	Key Limitation vs. Present Work
[12]	MRI → CT, PET → CT, CBCT → CT (general)	CNN, GAN	Minor	Not covered	Image quality metrics	Broad multimodality scope; no diffusion or conditional methods.
[13]	MRI → CT, CBCT → CT, PET AC	CNN, GAN, hybrid	Partial	Brief mention	Image + dose metrics (varied)	Multi-modality scope; diffusion not emphasized.
[14]	CBCT → CT (systematic review)	CNN, GAN, transformer	Primary	Early emerging	Image + dose metrics	Did not isolate conditional diffusion models.
(SynthRAD 2023, Report) [15]	MRI → CT, CBCT → CT (benchmark)	Multiple DL architectures	Benchmark subset	Evaluation only	Image–dose correlation	Benchmark paper, not a review; provides performance context.

**Table 2:** The PICO framework for systematic reviews.

**Population**	All patients underwent definitive oncology planning.
**Intervention**	Diffusion Model OR Conditional Diffusion OR Denoising Diffusion OR Score-Based Generative Model, CBCT OR Cone Beam Computed Tomography OR Cone-Beam CT, Imaging Reconstruction OR IR, Unsupervised Deep Learning OR UDL, Dose Estimations OR DE, Medical Imaging OR MI, Artifact Reduction OR AR, Radiotherapy.
**Comparison**	CT OR Computed Tomography.
**Outcome**	Sensitivity, Specificity, Accuracy.

**Table 3:** Synthesis of the included articles on sct generation using diffusion models.

Author/Year	Study Type	Population	Pre-Processing	Conditional Loss Function (Reverse Process)	Type of Neural Network (Reverse Process)	Training Strategies	Data Type	Outcomes (MAE, PSNR, etc.)	Noise Injection (Type and Levels)	Limitation	Findings
[18]	Frequency-Guided Diffusion Model (FGDM)	CBCT-CT datasets across institutions (various samples)	Frequency domain filtering (high-pass and low-pass)	Frequency-guided regularization	Freq.-guided diffusion model	Zero-shot domain adaptation	Paired and unpaired CBCT-C T data	FID improved, PSNR: 30+ dB	Controlled Gaussian noise	Limited robustness to freq. domain changes	Preserves structure in translation
[19]	Patient-specific model	Lung cancer (33 pts)	Normalization, fine-tuning	Fine-tuned with patient data	General lung diffusion model	Paired patient-specific training	Paired 2D slices	MAE: 15.96 HU, PSNR: 33.57 dB	Gaussian noise augmentation	Time-intensive tuning	Improved sCT quality, artifact correction
[20]	Patient-Specific Diffusion Model	Lung Cancer Patients (33 patients)	Anatomical fine-tuning	Custom fine-tuning loss	Patient-Specific DDPM	Paired fine-tuning per patient	Paired 2D slices	MAE: 15 HU, PSNR: 33 dB	Gaussian noise	High computation per patient	Effective sCT improvement
[21]	Diffusion model study	Chest Tumor Dataset (100+ samples)	Normalization	Energy-guided loss	UNet	Markov Chain Sampling	Unpaired 3D slices	MAE: 26.87 HU, PSNR: 19.83 dB	Gaussian noise	Mode collapse in GAN comparison	Superior to GAN-based methods
[11]	FGDM	CBCT-CT datasets across institutions (various)	Frequency domain analysis	Frequency-guided regularization	Frequency-guided diffusion mode	Zero-shot domain adaptation	Paired and unpaired CBCT-CT data	FID improved, PSNR: 30+ dB	Frequency-guided noise handling	Limited robustness to frequency domain changes	Preserves structural details during domain translation
[22]	Unsupervised Bayesian Framework	H&N, Lung, Pancreas (75 pts)	Score-based patient-specific priors	Total variation regularization	Patient-specific diffusion model	Unsupervised patient-specific training	Unpaired 3D slices	MAE: 50 HU, PSNR: 31 dB	Score-based noise scheduling	Slice alignment sensitivity	Effective artifact reduction
[7]	Conditional model	Brain, H&N (50 pts)	Cropping, normalization	L2 loss	Time-embedded UNet	Paired image data	Paired 2D slices	MAE: 25.99 HU, PSNR: 30.49 dB	Gaussian noise (time steps)	Requires large paired data	Improved CBCT quality for ART
[23]	Stacked coarse-to-fine	Pelvic cancer (250 pts)	Multi-stage denoising	Edge-preserving loss	DDPM with U-ConvNeXt	Hierarch training	Paired CBCT-CT slices	PSNR: 34.02 dB, SSIM: 87.14%	Hierarchical denoising	Paired data dependency	Enhanced ART dosimetric accuracy
[24]	Dual Energy Synthesis	H&N (54 pts)	CBCT-DECT normalization	Gradient matching loss	Multi-decoder Swin-UNET	Multi-decoder learning	Paired CBCT-DECT slices	MAE: 39.58 HU, PSNR improved	Controlled Gaussian noise	Dual-energy data complexity	Improved tissue characterization
[25]	Latent diffusion model	Prostate cancer (30 pts)	FFT-based high-freq extraction	Hybrid condition loss	Unified feature encoder	Hybrid high-freq embedding	Paired 3D slices	Gamma Passing Rate: 93.8%	Gaussian noise with FFT	Computation inefficiency	Enhanced anatomical preservation
[26]	Texture-preserving model	Multicenter CBCT-CT (100+ pts)	FFT, wavelet transforms	Boundary-aware loss	Dual-branch attention model	High-freq optimization	Unpaired 3D volumes	MAE: 18.48 HU, PSNR: 33.07 dB	Adaptive high-freq noise handling	High compute demand	Superior texture preservation

## Data Availability

The data supporting this systematic review are available within the article and its Supplementary Materials. These materials include the database-specific search strategies, the extracted study-level dataset used for the descriptive synthesis and QUADAS-2 risk of bias (traffic lights). No formal meta-analytic code is provided because the revised manuscript does not include pooled quantitative analysis.

## Supplementary Materials

The following supporting information can be downloaded at: https://www.mdpi.com/article/10.3390/tomography12050064/s1, Table S1. Full database-specific search strings; Table S2: Full extracted dataset of included studies; Figure S1: QUADAS-2 domain-level risk-of-bias assessment for the included studies. Green symbols indicate low concern and red symbols indicate high concern across the four QUADAS-2 domains: patient selection (D1), index test (D2), reference standard (D3), and flow and timing (D4), together with the overall judgment for each study.

## Abbreviations

The following abbreviations are used in this manuscript:

CBCT	Cone Beam Computed Tomography
CBCT-to-CT	Cone Beam Computed Tomography to Computed Tomography
CDM	Conditional diffusion model
CFG	Classifier-free guidance
CNN	Convolutional neural network
CT	Computed tomography
DCT	Discrete cosine transform
DDIM	Denoising diffusion implicit model
DDPM	Denoising diffusion probabilistic model
DECT	Dual-energy computed tomography
DVH	Dose-volume histogram
FFT	Fast Fourier transform
FGDA	Frequency-guided diffusion approach
FGDM	Frequency-guided diffusion model
FiLM	Feature-wise linear modulation
GAN	Generative adversarial network
H&N	Head and neck
HU	Hounsfield unit
LDM	Latent diffusion model
MAE	Mean absolute error
MRI	Magnetic resonance imaging
PICO	Population, Intervention, Comparison, Outcome
PET	Positron emission tomography
PRISMA	Preferred Reporting Items for Systematic Reviews and Meta-Analyses
PROSPERO	International Prospective Register of Systematic Reviews
PSNR	Peak signal-to-noise ratio
QUADAS-2	Quality Assessment of Diagnostic Accuracy Studies-2
sCT	Synthetic computed tomography
SSIM	Structural similarity index measure
VAE	Variational autoencoder
WoS	Web of Science
