# Supplementary material for: Conditional Diffusion Models for CT Image Synthesis from CBCT: A Systematic Review
Source: Tomography. 2026 May 6;12(5):64. doi: 10.3390/tomography12050064 (PMC13210980; doi:10.3390/tomography12050064)
Supplement: Supplementary file 1 [file tomography-12-00064-s001.zip › tomography-4213675-Supplementary.pdf]

# Supplementary Materials: Conditional Diffusion Models for CT Image Synthesis from CBCT: A Systematic Review

**Alzahra Altalib [1,2,*], Chunhui Li [1] and Alessandro Perelli [3]**

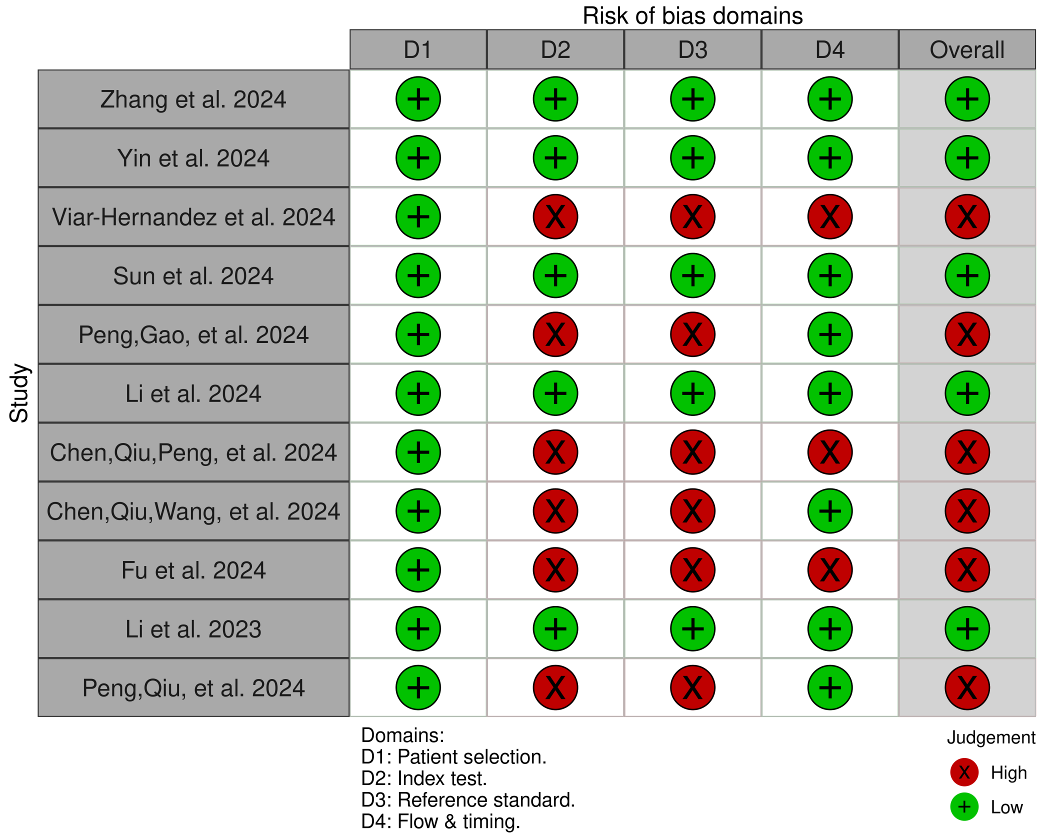


**Figure S1.** QUADAS-2 domain-level risk-of-bias assessment for the included studies. Green symbols indicate low concern and red symbols indicate high concern across the four QUADAS-2 domains: patient selection (D1), index test (D2), reference standard (D3), and flow and timing (D4), together with the overall judgment for each study.

**Table S1.** Full database-specific search strings.

| Database | Platform / field | Proposed search string | Filters / notes |
|---|---|---|---|
| PubMed | Title/Abstract | (("cone-beam computed tomography"[Title/Abstract] OR "cone beam computed tomography"[Title/Abstract] OR "cone-beam CT"[Title/Abstract] OR CBCT[Title/Abstract]) AND ("synthetic CT"[Title/Abstract] OR sCT[Title/Abstract] OR "CT synthesis"[Title/Abstract] OR "CBCT-to-CT"[Title/Abstract] OR "CBCT to CT"[Title/Abstract]) AND ("diffusion model*"[Title/Abstract] OR "conditional diffusion"[Title/Abstract] OR "denoising diffusion"[Title/Abstract] OR DDPM[Title/Abstract] OR DDIM[Title/Abstract] OR "latent diffusion"[Title/Abstract] OR "score-based"[Title/Abstract] OR "score based"[Title/Abstract])) | English; 2013–2024; last searched [insert date] |
| Web of Science | Topic search | TS=(("cone-beam computed tomography" OR "cone beam computed tomography" OR "cone-beam CT" OR CBCT) AND ("synthetic CT" OR sCT OR "CT synthesis" OR "CBCT-to-CT" OR "CBCT to CT") AND ("diffusion model*" OR "conditional diffusion" OR "denoising diffusion" OR DDPM OR DDIM OR "latent diffusion" OR "score-based" OR "score based")) | English; 2013–2024; indexes searched should be stated |
| Scopus | Title / Abstract / Keywords | TITLE-ABS-KEY ( ("cone-beam computed tomography" OR "cone beam computed tomography" OR "cone-beam CT" OR CBCT) AND ("synthetic CT" OR sCT OR "CT synthesis" OR "CBCT-to-CT" OR "CBCT to CT") AND ("diffusion model*" OR "conditional diffusion" OR "denoising diffusion" OR DDPM OR DDIM OR "latent diffusion" OR "score-based" OR "score based") ) | English; 2013–2024; last searched [insert date] |
| IEEE Xplore | Metadata / Abstract | (("All Metadata":"cone-beam CT" OR "All Metadata":"cone beam computed tomography" OR "All Metadata":CBCT) AND ("All Metadata":"synthetic CT" OR "All Metadata":"CBCT-to-CT" OR "All Metadata":"CT synthesis") AND ("All Metadata":"diffusion model" OR "All Metadata":"conditional diffusion" OR "All Metadata":"denoising diffusion" OR "All Metadata":"latent diffusion" OR "All Metadata":"score-based")) | English; 2013–2024; include conference proceedings if eligible |
| Google Scholar | Broad search | ("cone-beam CT" OR CBCT) AND ("synthetic CT" OR "CBCT-to-CT") AND ("diffusion model" OR "conditional diffusion" OR "denoising diffusion") | Screen by relevance and/or date; report how many records were screened manually |

**Table S2.** Full extracted dataset of included studies.

| Ref. | Study / shorthand | Publication status | Anatomy / population | Sample size | Paired / unpaired | Dimensionality | Diffusion/model family | Conditioning / guidance | Main reported outcomes | Key limitation | Key finding |
|---|---|---|---|---|---|---|---|---|---|---|---|
| [18] | Frequency-guided diffusion model | Conference abstract / meeting proceeding | Mixed CBCT-CT datasets across institutions | Various | Paired and unpaired | Not clearly reported | FGDM | Frequency-domain filtering / regularization | FID improved; PSNR 30+ dB | Limited robustness to frequency-domain changes | Preserved structure in translation |
| [19] | Patient-specific model | Peer-reviewed journal | Lung cancer | 33 pts | Paired | 2D slices | General lung diffusion model | Fine-tuning with patient data | MAE 15.96 HU; PSNR 33.57 dB | Time-intensive tuning | Improved sCT quality and artefact correction |
| [20] | Patient-specific diffusion model | Peer-reviewed journal | Lung cancer | 33 patients | Paired | 2D slices | Patient-specific DDPM | Anatomical fine-tuning | MAE 15 HU; PSNR 33 dB | High computation per patient | Effective sCT improvement |
| [21] | Energy-guided diffusion model | Peer-reviewed journal | Chest tumour dataset | 100+ samples | Unpaired | 3D slices | UNet Markov-chain sampling | Energy-guided loss | MAE 26.87 HU; PSNR 19.83 dB | Mode collapse in GAN comparison | Superior to GAN-based methods in that study context |
| [11] | Zero-shot FGDM | Peer-reviewed journal | Mixed CBCT-CT datasets across institutions | Various | Paired and unpaired | Not clearly reported | Frequency-guided diffusion | Frequency-domain analysis / regularization | FID improved; PSNR 30+ dB | Limited robustness to frequency-domain changes | Preserved structural detail during domain translation |
| [22] | Unsupervised Bayesian framework | Preprint | H&N, lung, pancreas | 75 pts | Unpaired | 3D slices | Patient-specific diffusion / score-based prior | Score-based patient-specific priors | MAE 50 HU; PSNR 31 dB | Slice alignment sensitivity | Effective artefact reduction |
| [7] | Conditional DDPM model | Peer-reviewed journal | Brain, H&N | 50 pts | Paired | 2D slices | Time-embedded UNet | L2 loss / paired image conditioning | MAE 25.99 HU; PSNR 30.49 dB | Requires large paired data | Improved CBCT quality for ART |
| [23] | Stacked coarse-to-fine model | Peer-reviewed journal | Pelvic cancer | 250 pts | Paired | Slice-based | DDPM with U-ConvNeXt | Edge-preserving loss / hierarchical denoising | PSNR 34.02 dB; SSIM 87.14% | Paired data dependency | Enhanced ART dosimetric accuracy |
| [24] | Dual-energy synthesis | Peer-reviewed journal | H&N | 54 pts | Paired | Slice-based | Multi-decoder Swin-UNET | Gradient-matching loss / CBCT-DECT normalization | MAE 39.58 HU; PSNR improved | Dual-energy data complexity | Improved tissue characterization |
| [25] | HC3 L-Diff | Preprint | Prostate cancer | 30 pts | Paired | Latent / 3D | Latent diffusion model | Hybrid conditional loss / FFT high-frequency enhancement | Gamma passing rate 93.8% | Computational inefficiency | Enhanced anatomical preservation |
| [26] | Texture-preserving diffusion model | Peer-reviewed journal | Multicentre CBCT-CT | 100+ pts | Unpaired | 3D volumes | Dual-branch attention diffusion | Boundary-aware loss / FFT + wavelets | MAE 18.48 HU; PSNR 33.07 dB | High compute demand | Superior texture preservation |
